# Antagonism between FOXO and MYC Regulates Cellular Powerhouse

**DOI:** 10.3389/fonc.2013.00096

**Published:** 2013-04-25

**Authors:** Barrie Peck, Emma C. Ferber, Almut Schulze

**Affiliations:** ^1^Gene Expression Analysis Laboratory, Cancer Research UK, London Research InstituteLondon, UK

**Keywords:** FOXO, MYC, ROS, hypoxia, metabolism, mitochondria, HIF, cancer

## Abstract

Alterations in cellular metabolism are a key feature of the transformed phenotype. Enhanced macromolecule synthesis is a prerequisite for rapid proliferation but may also contribute to induction of angiogenesis, metastasis formation, and tumor progression, thereby leading to a poorer clinical outcome. Metabolic adaptations enable cancer cells to survive in suboptimal growth conditions, such as the limited supply of nutrient and oxygen often found in the tumor microenvironment. Metabolic changes, including activation of glycolysis and inhibition of mitochondrial ATP production, are induced under hypoxia to promote survival in low oxygen. FOXO3a, a transcription factor that is inhibited by the phosphatidylinositol 3-kinase/Akt pathway and is upregulated in hypoxia, has emerged as an important negative regulator of MYC function. Recent studies have revealed that FOXO3a acts as a negative regulator of mitochondrial function through inhibition of MYC. Ablation of FOXO3a prevents the inhibition of mitochondrial function induced by hypoxia and results in enhanced oxidative stress. This review will focus on the antagonism between FOXO3a and MYC and discuss their role in cellular bioenergetics, reactive oxygen metabolism, and adaptation to hypoxia, raising questions about the role of FOXO proteins in cancer.

## Introduction

The process of malignant transformation that cells undergo to form cancers has been the focus of cancer research since its inception. The “hallmarks of cancer” were originally designated as self-sufficiency in growth signals, insensitivity to growth inhibitory signals, unlimited replicative potential, evasion of programed cell death, sustained angiogenesis, tissue invasion, and metastasis (Hanahan and Weinberg, 2011). More recently, additional hallmarks have been added, including evasion from destruction by the immune response, genome instability, the role of the microenvironment, pro-tumorigenic inflammation, and deregulation of cellular energetics (Hanahan and Weinberg, 2011).

The altered metabolism of tumor cells is not a new concept in cancer biology. In the early 1920s, Otto Warburg established that cancerous tissues have an altered metabolism, compared to their non-tumorigenic counterparts and satisfy their bioenergetic demands by shifting from oxidative phosphorylation (OXPHOS) to glycolysis. Although this shift decreases the efficiency of ATP production per molecule of glucose, it favors the shuttling of metabolic intermediates to biosynthetic processes required for macromolecule biosynthesis (Vander Heiden et al., 2009). The increased metabolic activity of cancer cells results in increased cellular stress through the generation of reactive oxidative species (ROS) – a cellular milieu of highly reactive oxygen ions and peroxides – which can both impact on cell survival and transformation. ROS are known to damage proteins and DNA, promote cellular aging and, as a consequence, induce the onset of age-related diseases including cancer (Benz and Yau, 2008). However, maintenance of cellular ROS is indispensible for the pro-tumorigenic capacity of some oncogenes such as RAS (De Raedt et al., 2011). Moreover, high ROS lead to the stabilization of hypoxia inducible factors (HIFs) by inhibiting the activity of prolyl hydroxylases (PHDs) that are involved in the oxygen-dependent destabilization of HIF proteins (Kaelin and Ratcliffe, 2008). HIFs are important factors that facilitate the metabolic adaption of cells to low oxygen (hypoxic) conditions. Hypoxic regions are a common feature of many solid tumors and the induction of tumor hypoxia is positively correlated with a poorer clinical outcome (Harris, 2002). Thus, the role of ROS in cancer is complex and elucidating the mechanisms by which the redox balance is regulated is critical to understanding cancer cell biology and the development of strategies for successful treatments.

## Forkhead Transcription Factors

The phosphatidylinositol 3-kinase (PI3K) signaling pathway consists of enzymes and phospholipid second messengers that are involved in signal transduction and regulate cellular functions, such as motility, cell growth, proliferation, differentiation, survival, and apoptosis. The PI3K cascade is frequently hyper-activated in human cancers. Dysregulation of its components, such as PTEN, PIK3CA, and AKT (PKB), is common in solid tumors including breast, colon, endometrial, and prostate cancers (Samuels et al., 2004; Zhao and Vogt, 2008; Dillon and Muller, 2010; Song and Salmena, 2012). Established oncogenic signaling pathways such as the PI3K/AKT cascade are known to regulate metabolic processes via discrete transcriptional effectors, such as the sterol regulatory element binding proteins (SREBPs) and forkhead transcription factors of the O-class (FOXOs).

Forkhead box proteins belong to a large family of transcription factors, which share an evolutionarily conserved DNA-binding domain (DBD) (Weigel et al., 1989; Burgering, 2008). Forkhead proteins are divided into 19 subgroups, each class termed A-S, based on their sequence similarity and structure, not function (Kaestner et al., 2000). Outside of the DBD, forkhead proteins vary significantly.

Mammalian FOXO proteins are orthologs of the transcription factor DAF-16 (abnormal DAuer Formation 16) identified in the nematode *Caenorhabditis elegans* and found to be a component of metabolic insulin signaling and longevity (van der Horst and Burgering, 2007). The FOXO class of proteins consists of four members, FOXO1, FOXO3a, FOXO4, and FOXO6, which are negatively regulated by the PI3K signaling cascade effector protein AKT (Brunet et al., 1999). When PI3K/AKT signaling is activated, FOXO proteins are phosphorylated by AKT on discrete residues that lead to its inactivation and exclusion from the nucleus. FOXO1, 3, and 4 are ubiquitously expressed, although FOXO1 is expressed more highly in adipose tissues. FOXO3a is expressed highly in the liver and FOXO4 is highly expressed in skeletal muscle. FOXO6 is predominantly found in the brain (Burgering, 2008; Fu and Tindall, 2008).

**Figure 1:**
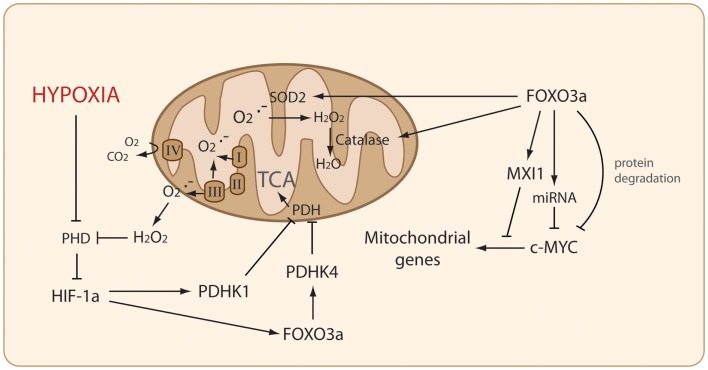
**FOXO3a modulates both ROS production and detoxification**. FOXO3a inhibits Reactive Oxidative Species (ROS) production through several mechanisms to perturb the MYC-dependent expression of nuclearly encoded mitochondrial genes. FOXO3a mediates ROS detoxification by upregulating the expression of superoxide dismutase (SOD2) and Catalase. Under hypoxia, inhibition of Prolyl hydroxylases (PHDs) results in HIF-1a activation, which upregulates several target genes including PDHK1 and FOXO3a – which in turn upregulates PDHK4 – to coordinately inhibit pyruvate dehydrogenase (PDH).

FOXO proteins bind to the consensus motif (daf-16 binding element – DBE) 5′-TTGTTTAC-3′ within the target gene promoter via their DBD (Furuyama et al., 2000; van der Horst and Burgering, 2007). Once bound, the C-terminal transactivation domain initiates gene transcription. FOXO proteins have a plethora of transcriptional targets involved in several cellular processes, including cell cycle arrest (Medema et al., 2000, p. 27), DNA damage [GADD45 (Tran et al., 2002)], cell signaling [HER3 (Chandarlapaty et al., 2011)], and apoptosis [BIM (Stahl et al., 2002)]. An important function of FOXO proteins is their control of the cellular redox balance; indeed, the transcriptional activity of FOXO4 is influenced by redox-sensitive cysteine residues that increase its interaction with the histone acetyltransferase p300 in the presence of high ROS (Dansen et al., 2009). The induction of ROS detoxifying mechanisms through downstream targets of FOXOs, such as superoxide dismutase (SOD2) and catalase, has been maintained throughout evolution. In mammalian cells, SOD2 and catalase remove mitochondrial superoxide and hydrogen peroxide, respectively (Kops et al., 2002). Increased ROS detoxification promotes cell survival in unfavorable redox environments. More recently, two studies have shown that FOXO activation reduces ROS production by decreasing mitochondrial function through inhibition of MYC (Jensen et al., 2011; Ferber et al., 2012) (Figure 1).

## MYC

c-MYC (MYC) is a transcription factor which binds to an Enhancer box (E-box) consensus sequence within the promoter regions of target genes (Dang et al., 2006). MYC is known to regulate the expression of 15% of all genes in the human genome and is an important regulator of cell proliferation (Dang, 2012a). Furthermore, MYC amplifies gene expression of pre-selected genes, rather than initiating transcription (McCarthy, 2012). In normal cells MYC is an important driver of bioenergetic processes whose activity is tightly regulated by growth factor availability, facilitating proliferation only in a favorable environment (Dang, 2012b). Constitutive activation of MYC allows transformed cells to proliferate independently of growth promoting signals. MYC is one of the most studied proto-oncogenes in cancer to date and as many as 70% of tumors are thought to display MYC overexpression, either through increased gene copy number or mRNA overexpression (Gordan et al., 2007). MYC overexpression drives cell cycle progression by activating key components of the cell cycle machinery [Cyclins and cyclin-dependent kinases (CDKs)] and by inhibiting the expression of CDK inhibitors (Meyer and Penn, 2008). Also, MYC overexpression is responsible for several metabolic changes in cancer. MYC promotes glutaminolysis by driving the expression of several enzymes within this pathway. This includes glutaminase (GLS), the enzyme that converts glutamine to glutamate, which is then converted to alpha-ketoglutarate by glutamine dehydrogenase (GLDH) and subsequently enters the tricarboxylic acid (TCA) cycle to produce oxaloacetate (Wallace, 2012). The activation of glutaminolysis by MYC results in a strong dependency of cancer cells on glutamine (glutamine addiction). Furthermore, MYC is a major regulator of mitochondrial biogenesis through the activation of genes such as mitochondrial transcription factor A (TFAM) (Li et al., 2005) and PGC-1-β (PPARGC1B) (Zhang et al., 2007).

## FOXOs Antagonism of MYC

The antagonism of MYC by FOXO was initially identified in murine B cell lymphoma cells. Ectopic expression of MYC is able to block FOXO-dependent transcription of p27 in response to pharmacological inhibition of the PI3K cascade (Chandramohan et al., 2004). A number of studies have highlighted the multiple mechanisms through which FOXO proteins antagonize MYC function. Activation of FOXO factors following inhibition of PI3K/AKT signaling inhibits MYC target genes by directly binding to their promoters (Bouchard et al., 2004). Delpuech et al. (2007) showed that the MAX-interacting protein (MXD) family members are key downstream effectors of FOXO3a and are involved in its anti-proliferative function. FOXO3a binds directly to the promoter of MAX-interacting protein 1 (MXI1) and activates its expression while other family members are upregulated through an indirect mechanism. MXD family proteins antagonize MYC function by binding to promoter regions of MYC target genes as heterodimers with the MYC-associated factor X (MAX), an essential component of the transcriptionally active MYC/MAX heterodimer, thus preventing MYC from binding and expediting gene transcription (Delpuech et al., 2007). Subsequent studies have shown that FOXO3a can also inhibit MYC activity by increasing the expression of micro-RNAs (miRNAs) that perturb the translation of the MYC mRNA (Gan et al., 2010; Kress et al., 2011; Ferber et al., 2012). Genome-wide ChIP-seq of FOXO3a also supports the notion of an inverse correlation between FOXO3a and MYC function (Eijkelenboom et al., 2013), although MYC is able to displace FOXO from the promotor of its downstream targets, GADD45 and PUMA (Amente et al., 2011), suggesting there is some reciprocal regulation of FOXO3a activity by MYC.

Two recent studies have demonstrated that the inhibition of nuclearly encoded mitochondrial genes by FOXO3a involves inhibition of MYC though multiple mechanisms (Figure 1). Induction of MAD/MXD proteins by FOXO3a is required for efficient inhibition of these genes. In addition, FOXO3a activation reduces MYC protein stability by increasing the phosphorylation of the MYC phosphodegron motif (Ferber et al., 2012), which mediates the degradation of MYC via the SCF/FBW7 ubiquitin system. Controlling MYC at multiple levels may enable FOXO proteins to deliver different temporal effects on mitochondrial output. Acute inhibition of mitochondrial gene expression could be achieved by rapidly decreasing MYC protein levels by reducing its stability and inhibiting the translation of its mRNA, but sustained inhibition may be maintained through changes in promoter occupancy. Both studies investigated the regulation of mitochondria by FOXO and demonstrated that FOXO3a decreases ROS levels. This was independent of previously described FOXO3a-induced expression of ROS detoxification genes, but required MYC inhibition (Jensen et al., 2011; Ferber et al., 2012). Regulating cellular ROS levels is critically important for many cellular functions. While high ROS levels induce cell damage leading to apoptosis and cell death, controlled ROS production has an important role in signaling that can determine cell fate and promote cell transformation and cancer development (Hamanaka and Chandel, 2010).

## FOXO and Hypoxia

The proliferative celerity of an expanding tumor imparts substantial pressure on its microenvironment by the increased demand of nutrients and oxygen. The surrounding vasculature is often unable to fulfill this demand, resulting in regions of nutrient and oxygen deprivation. The formation of hypoxic regions within the tumor initiates certain adaptations within the cancer cell metabolism. These adaptations involve a shift from oxidative metabolism to an increased dependency on glycolysis.

Induction of the hypoxia inducible factor 1 alpha (HIF-1a) in low oxygen increases the transcription of glycolytic enzymes and induces ATP generation from glycolysis. HIF-1a induces the expression of pyruvate dehydrogenase kinase (PDHK1) inhibiting pyruvate dehydrogenase (PDH) activity, thereby blocking pyruvate decarboxylation to Acetyl-CoA and entry into the tricarboxylic acid (TCA) cycle. This decreases mitochondrial respiration and ATP production by OXPHOS. The accumulating pyruvate is converted into lactate by lactate dehydrogenase (LDH), which recycles NAD+ from NADH to sustain the increased glycolytic flux (Simon, 2006). HIF expression is key to adaption and survival under hypoxia. However, HIF activation also needs to be restrained, as HIF-1a is known to induce apoptosis via p53-dependent and independent mechanisms following chronic hypoxia (Harris, 2002). In this regard, Jensen et al. (2011) show that FOXO3a is activated *in vivo* in hypoxic tumor tissue and that elevated ROS following FOXO3a silencing sensitizes cells to hypoxia-induced cell death. Moreover, FOXO3a silencing slowed tumor growth in xenografts *in vivo* and the tumors displayed increased caspase-3 staining indicating apoptosis. The resulting tumors showed increased expression of mitochondrial genes and a decreased rate of glucose uptake, indicating increased mitochondrial metabolism. Therefore, changing the metabolic capacity of cancer cells within a solid tumor may enhance their short-term proliferative potential but compromise their long-term survival, as the cancer cells are unsuitably adapted to their environment. The study by Ferber et al. (2012) demonstrates that reduced mitochondrial ROS production in response to FOXO3a activation has an important role in hypoxic signaling. Under hypoxia, the absence of oxygen as an electron acceptor causes a burst of superoxide production produced by electron leakage within complexes I and III of the respiratory chain. While complex I-derived superoxide is mainly retained within the mitochondrial matrix, superoxide produced by complex III is released into the cytoplasm. Cytoplasmic superoxide is required for HIF-1a stabilization as PHDs are inhibited by ROS (Klimova, 2008). FOXO3a blocks the hypoxia-induced ROS increase, thereby preventing HIF-1a stabilization (Figure 1). Importantly, HIF-1a levels in hypoxia following FOXO3a activation are rescued by restoring MYC expression, or by addition of exogenous oxidant, showing that perturbing MYC function is central to FOXO3a’s regulation of HIF-1a (Ferber et al., 2012). Silencing of endogenous FOXO1 or FOXO3a in hypoxia resulted in a de-repression of mitochondrial gene expression, although MXI1 expression remained unaffected (Ferber et al., 2012). This is in agreement with a study by Zhang et al. (2007) demonstrating that HIF-1a is responsible for driving MXI1 expression in hypoxia. Predominantly, HIF-1a limits MYC activity and proliferation in hypoxia but this antagonism is lost when MYC is highly overexpressed. In this context HIF-1a and MYC cooperate to regulate the expression of PDHK1, hexokinase 2 (HK2), and vascular endothelial growth factor A (VEGFA). Another HIF isoform, HIF-2a, cooperates with MYC to drive tumorigenesis. HIF-2a binds to MAX, promoting its interaction with MYC, increasing its activity (reviewed in Keith et al., 2012).

FOXO3a is also able to modulate MYC transcriptional activity by additional mechanisms to reduce ROS production in hypoxic cells. Bakker et al. (2007) have shown that FOXO3a is activated in low oxygen and constrains HIF-1a activity by increasing the expression of CITED2, a negative regulator of HIF-1a. By downregulating mitochondrial gene expression, as well as mitochondrial activity, FOXO3a replaces some of the functions of HIF-1a in the cellular adaptation to hypoxia. However, by preventing HIF-1a stabilization FOXO3a could also act to hijack the cellular response to low oxygen and facilitate other cellular outcomes. FOXO3a-dependent repression of mitochondrial function could provide protection from pro-apoptotic stimuli under conditions of chronic hypoxia, and as such, affect tumorigenesis and cancer progression.

## FOXOs in Cancer

Limiting mitochondrial ROS production may impact on cancer biology in multiple ways. Since mitochondrial ROS contribute to RAS-dependent tumorigenesis (Weinberg et al., 2010), inhibition of mitochondrial activity by FOXO proteins could be an important aspect of their function as tumor suppressors. However, by decreasing the sensitivity of tumor cells to apoptosis, FOXO proteins may promote cell survival under hypoxia, and enhance tumor growth. This suggests a potential oncogenic role for FOXO in cancer progression. The effect of the inhibition of mitochondrial activity by FOXO proteins on tumorigenesis is likely to be context dependent with overall ROS levels critical in determining cancer cell fate. In fact, FOXOs role as a determinant of cell fate has been demonstrated in the hematopoietic system, where FOXO3a-dependent ROS regulation promotes the long-term maintenance of the hematopoietic stem cell (HSC) pool (Miyamoto et al., 2007). A number of reports have shown that FOXOs are important for the maintenance of self-renewal capacity in normal HSCs and neural stem cells (NSCs) (Tothova and Gilliland, 2007; Tothova et al., 2007; Paik et al., 2009; Renault et al., 2009). The ability of FOXO to decrease ROS is required to maintain stem cell quiescence and preserve long-term self-renewal capacity. In HSCs, *FoxO*-deficiency leads to defective long-term repopulating activity, correlated with increased cell proliferation and apoptosis and accompanied by increased ROS levels and expression of ROS-regulating genes. Treatment with the antioxidant *N*-acetyl-l-cysteine (NAC) rescued the effect of *FoxO* loss in the bone marrow by reinstating HSC’s self-renewal capacity (Tothova et al., 2007). Interestingly, this function is maintained in certain leukemias; Naka et al. (2010) showed that in chronic myeloid leukemia (CML) FOXO signaling plays a role in maintaining leukemia-initiating cells (LIC) – the population responsible for the development of recurrent disease. In these cells, AKT activity is suppressed by TGF-beta, despite the expression of the BCR-ABL fusion protein, leading to nuclear localization and activation of FOXO3a, maintaining their survival. FOXO3a was also required for the colony forming capacity of the LICs, which could be reversed with combinatorial treatment using the BCR-ABL inhibitor Imatinib and the TGF-Beta inhibitor LY364947 (Sykes et al., 2011). This led the authors to speculate that inhibition of FOXO function could be considered for the treatment of CML. Strikingly, in acute myeloid leukemia (AML) approximately 40% of patient samples displayed elevated FOXO activity (Sykes et al., 2011). This increased activity maintained leukemic growth by preventing myeloid maturation and apoptosis. Combined inhibition of FOXOs by either deletion of the gene or by activation of AKT *in vivo*, improved survival (Sykes et al., 2011).

The vital role of FOXOs in controlling ROS levels and their involvement in HSC maintenance also has potential implications for the development of solid tumors. The juxtaposition of FOXO factors downstream of several important signaling cascades, and the large number of transcriptional targets, make it hard to pinpoint their exact contribution to cancer development. Although it has been extensively shown that FOXO activation inhibits the proliferative potential of cancer cells in solid tumors, how and whether it also alters their capacity to differentiate or acquire stem cell characteristics, is yet to be fully deduced. In this regard, FOXOs can be considered as two-faced characters within the transformation process: they inhibit proliferation in some cancer cells while promoting survival in others. It is likely that the response to FOXO activation is determined by the different genetic backgrounds of cancer cells, but also affected by the microenvironment. Tenbaum et al. (2012) have shown that Beta-catenin is able to subvert FOXO3a function in colon cancer cells, inhibiting the transcription of pro-apoptotic genes, while activating a transcriptional program that promotes metastasis. The heterogeneous expression of Beta-Catenin throughout the tumor, results in apoptosis following treatment with PI3K/AKT inhibitors, but only in cell populations that express low levels of Beta-Catenin. In contrast, PI3K/AKT inhibitors exacerbate the metastatic potential of cancer cells expressing high levels of Beta-Catenin. Crucially, co-expression of Beta-Catenin and FOXO3a in late-stage cancers was associated with a poorer clinical outcome (Tenbaum et al., 2012). The nuclear localization of FOXO3a predominantly indicates a good prognosis (Habashy et al., 2011), although a recent study showed that increased nuclear FOXO3a staining correlated with decreased survival in breast cancer patients (Chen et al., 2010).

## Future Perspectives

The complexity of the antagonistic interaction between FOXO factors and MYC underscores their significance as important regulators of cell metabolism, proliferation, and survival. However, more work is needed to understand the dynamic regulation of FOXO proteins, whose subcellular localization and transcriptional activity are regulated by several signaling pathways that play an important role in cancer. These include the canonical PI3K/AKT pathway, but also the RAS/MEK/ERK signaling axis (Yang et al., 2008), the IKKBeta/NF-kappaB pathway (Hu et al., 2004) and the Wnt signaling network (Dehner et al., 2008). Understanding the upstream inputs that determine FOXO function is essential for the development of appropriate strategies to target it in cancer. It is tempting to speculate that therapies that induce and maintain FOXO3a-dependent MYC inhibition could have potent anti-cancer effects, at least during early tumorigenesis. But given the role of FOXOs in ROS detoxification and the maintenance of cellular redox balance, it is possible that the same therapies could exacerbate tumor burden during later stages of the disease.

The therapeutic impact of targeting MYC in cancer is substantial (Soucek et al., 2008); but efforts to pharmacologically inhibit MYC to date have been variable. Recently, focus has been aimed at identifying synthetic lethal interactions associated with MYC overexpression and the indirect inhibition of MYC function via interacting co-factors (Reviewed in Prochownik and Vogt, 2010). Compounds that inhibit MYC activity by blocking MAX binding or stabilize MAX homodimerization have been identified and shown to be efficacious in *in vitro* assays and in cancer cell lines (Jiang et al., 2009). Indeed, curbing MYC activity is central to the control of proliferation by the PI3K signaling cascade. Liu et al. have shown that MYC amplification led to growth independent of the PI3K cascade and resistance to PI3K inhibition. The dual targeting of components of the PI3K cascade and MYC was required to circumvent resistance (Liu et al., 2011).

Mitochondrial integrity and efficiency is important for the regulation of lifespan and the onset of age-related diseases. FOXOs role in regulating ROS is therefore important in the wider context of cellular aging and organismal lifespan. Studies have shown that homologs of FOXO3a influence lifespan in model organisms, and are indispensible for lifespan extension in response to caloric restriction. Interestingly, studies have identified single nucleotide polymorphisms (SNPs) in FOXO3a that are associated with longevity in several geographically diverse human populations (Willcox et al., 2008; Flachsbart et al., 2009; Li et al., 2009).

Together these findings establish FOXOs as major regulators of cellular redox balance with major implications for human health, including aging and cancer. The full repertoire of tools that FOXOs employ to regulate these multiple functions however, is not completely defined. The jury is still out on the sly old FOX(O).

## Conflict of Interest Statement

The authors declare that the research was conducted in the absence of any commercial or financial relationships that could be construed as a potential conflict of interest.
